# Indiana Association of Veterinary Graduates

**Published:** 1895-10

**Authors:** J. C. Rodger

**Affiliations:** Secretary


### SOCIETY PROCEEDINGS.

### INDIANA ASSOCIATION OF VETERINARY GRADUATES.

THE semi-annual meeting was held in the parlors of the Hotel Burrier, Marion, July 9 and 10, 1895, Dr. J. E. Cloud, Richmond, in the chair. Members present: C. F. Bell, Kokomo; F. A. Bolser, New Castle; J. W. Klotz, Noblesville; W. B. Wallace, Marion; F. W. Anderman, Hartford City; O. L. Boor, Muncie; J. C. Rodger, Anderson; Visitor, G. L. Simon, Marion.

The minutes of the previous meeting were read and approved. On motion Dr. G. L. Simon was proposed and elected to membership. The Committee on Legislation made their report and were continued. A paper was then read by Dr. J. W. Klotz on "Ovariotomy," which was well received by the members, and a good discussion followed.

Adjourned until 9 A.M., July 10th. At the hour named the meeting was called to order by the President in the chair. A paper was then read by Dr. R. F. Bell, written by Dr. H. R. Macaulay, of Indianapolis (who was absent), on "Pleurisy and its Sequels," which was well written, and showed that the writer was familiar with the diseases of the lungs. A good discussion followed. On motion the resignation of Dr. J. H. Honan, of Hammond, was accepted, as the doctor having graduated from a Chicago medical college had decided to practise on the human subject, all the members wishing him success in his new sphere. On motion the resignation of Ex-State Veterinarian C. M. Stull was accepted, as the doctor is thinking of changing his professional card. A vote of thanks was tendered Dr. W. B. Wallace for the kindly manner in which he had entertained the members of the Association. On motion the meeting adjourned to meet in Muncie, December 10 and 11, 1895.

After adjournment the members were treated to a dinner at one of the leading cafés in the city, consisting of horse-meat cooked in all the ways known to the caterers' art, which had been selected by Dr. Wallace months previous to our meeting, and kept in his infirmary to be noted that it was free from disease, after which the animal, a two-year-old, was slaughtered and served, and last but not least eaten with a relish and pronounced first-class, after which glasses were filled and drank to the health of Dr. Wallace, who had taken upon himself such a laborious task of feeding a lot of hungry vets.

J. C. RODGER,
Secretary.

